# Acoustically semitransparent nanofibrous meshes appraised by high signal-to-noise-ratio MEMS microphones

**DOI:** 10.1038/s44172-024-00283-4

**Published:** 2024-09-23

**Authors:** Hutomo Suryo Wasisto, Sebastian Anzinger, Giovanni Acanfora, Aloysius Farrel, Valentina Sabatini, Elisa Grimoldi, Vasco Marelli, Nikita Ovsiannikov, Konstantin Tkachuk, Giordano Tosolini, Carmine Lucignano, Marco Mietta, Guangzhao Zhang, Marc Fueldner, Erwin Peiner

**Affiliations:** 1 Infineon Technologies AG, Am Campeon 1-15, Neubiberg, Germany; 2 Institute of Semiconductor Technology (IHT) and Laboratory for Emerging Nanometrology (LENA), Technische Universität Braunschweig, Hans-Sommer-Str. 66, Braunschweig, Germany; 3 TUM School of Natural Sciences, Technische Universität München, James-Franck-Str. 1, Garching, Germany; 4 SAATI SPA, Via Milano 14, Appiano Gentile, Como, Italy; 5 TUM School of Computation, Information, and Technology (CIT), Technische Universität München, Arcisstraße 21, München, Germany

**Keywords:** Sensors, Electrical and electronic engineering

## Abstract

Microelectromechanical system-based microphones demand high ingress protection levels with regard to their use in harsh environment. Here, we develop environmental protective components comprising polyimide nanofibers combined onto polyether ether ketone fabric meshes and subsequently appraise their impact on the electroacoustic properties of high signal-to-noise-ratio microelectromechanical system-based microphones via industry-standard characterizations and theoretical simulations. Being placed directly on top of the microphone sound port, the nanofiber mesh die-cut parts with an inner diameter of 1.4 mm result in signal-to-noise-ratio and insertion losses of (2.05 ± 0.16) dB(A) and (0.30 ± 0.11) dBFS, respectively, in electroacoustic measurements. Hence, a high signal-to-noise-ratio value of (70.05 ± 0.17) dB(A) can be maintained by the mesh-protected microphone system. Due to their high temperature stability, acoustic performance, environmental robustness, and industry-scale batch production, these nanofibrous meshes reveal high potential to be practically implemented in high-market-volume applications of packaged microelectromechanical system-based microphones.

## Abstract (web summary)

Hutomo Suryo Wasisto and colleagues develop a nanofiber-based mesh for improving the ingress protection level of microelectromechanical system (MEMS)-based microphone. Their device demonstrates high acoustic performance and environmental robustness.

## Introduction

Microelectromechanical system (MEMS)-based microphones have continuously been used in widespread applications due to their offered advantages (i.e., high and reproducible acoustic performance, high compatibility with application-specific integrated circuits (ASICs), good temperature stability, low manufacturing cost, and ability to be integrated into a small package via reflow soldering)^1–4^. Nowadays, high-volume MEMS microphones are not only integrated in consumer electronics (e.g., smartphones, tablets, smartwatches, laptops, and true-wireless earphones), but also extended to automotive, medical, and industrial devices^5,6^. Besides optimizations of key acoustic performance parameters (e.g., sensitivity, noise, and signal-to-noise ratio (SNR)) using either a true-differential MEMS transducer^7^ or novel membrane materials^8–10^, focus and efforts have been devoted to increase the robustness level of packaged MEMS microphones towards environmental influence from dust particles and liquid ingression^11^.

While the recently developed MEMS microphones from Infineon Technologies AG have already incorporated a basic ingress protection (IP) level to dust and water (IP57)^12^, particularly macro-scale impacting objects (e.g., sand grains, hairs, and metal swarf) still can damage their typically thin polysilicon membranes. Therefore, additional protective components (e.g., meshes or membranes) can be optionally included into the sound channels or housing of electronic devices (e.g., smartphones and smartwatches) to better protect the microphones from such interfering substances. However, the resulting higher protection level comes with adverse effects since adding a barrier in an acoustic channel will reduce the microphone sensitivity (insertion loss) and/or increase its noise, which can deteriorate the system SNR^13^. Moreover, nowadays, the employed environmental protective components for consumer electronics typically comprise per-and poly-fluoroalkyl substance (PFAS) materials, which are a class of fluorinated organic chemicals. These materials have been intensively discussed by many regulatory initiatives across different countries in e.g., Asia, Europe, and North America because their implementation to products may be restricted in the near future^14–16^. Thus, challenges remain at producing PFAS-free environmental protective components in industry-scale that can accommodate both high protection levels and excellent acoustic performance.

Nanoscale fibrous structures or nanofibers fabricated using electrospinning offer a multitude of advantageous features (e.g., excellent mechanical behavior, high surface-area-to-volume ratio, tunable surface morphology, and customizable porosity), and can be produced in large-scale using roll-to-roll processes^17,18^. These features make them particularly attractive for micro-/nanoparticle filtration purposes^19,20^. The corresponding electrospun nanofiber-based particle filters have not only been developed as prototypes by the universities, but also manufactured and commercialized by industrial companies (e.g., SAATI SPA). Besides particle filtration, electrospun nanofibers have been extensively explored and employed in other applications (e.g., tissue engineering scaffolds, gas sensors, humidity sensors, smart surfaces, wastewater purifier, energy storages, and safe engineering of therapeutic cells)^21–27^. To fabricate nanofiber-based particle filters, various polymers such as polyacrylonitrile (PAN), polyetherimide (PEI), polyvinyl alcohol (PVA), and polyimide (PI) can be opted to be either used alone resulting in pure nanofibers or combined with other materials (e.g., silver nanoparticles, ZIF-8, cellulose, or chitosan) to form composite nanofibers^19,20^.

Among those materials, PI nanofibers are favorable to be employed as protective components for acoustic sensing devices like MEMS microphones because of their outstanding properties (i.e., high mechanical strength, porosity, temperature stability, chemical resistance, and hydrophobicity)^28,29^. PI is a high-performance engineering polymer with macromolecular repeating units containing the functional imide group. Among the commercially available PI variants (i.e., aliphatic, semi-aromatic and aromatic PIs), the aromatic PI, which possesses heterocyclic imide rings and aromatic benzene rings in its macromolecular backbone, is the most appreciated due to its superior material properties^28,29^. Nonetheless, despite all those advantageous intrinsic material properties of the PI nanofibers, their impact on microphone-system performance (i.e., sensitivity, noise, and SNR) still needs to be investigated.

In this work, environmental protective components combining PI nanofibers with polyether ether ketone (PEEK) monofilament fabric meshes were developed and evaluated in terms of their environmental-robustness capability, acoustic properties, and integrability with high-SNR MEMS microphones in industry-standard setups. Simulations were performed for both the nanofibrous mesh and the MEMS microphone to estimate their combined acoustic system performance. Insertion and SNR losses were extracted from electroacoustic measurements and analyzed allowing further strategies to design high-performance acoustic-sensing devices. Lastly, various robustness tests (i.e., particle filtration, static pressure, hair drop, water-contact angle, and dust-particle tests) were carried out to investigate the robustness level of the developed nanofibrous meshes at both die-cut-part and microphone-system levels.

## Methods

### Polyimide nanofibrous meshes and die-cut parts

**Fig. 1:**
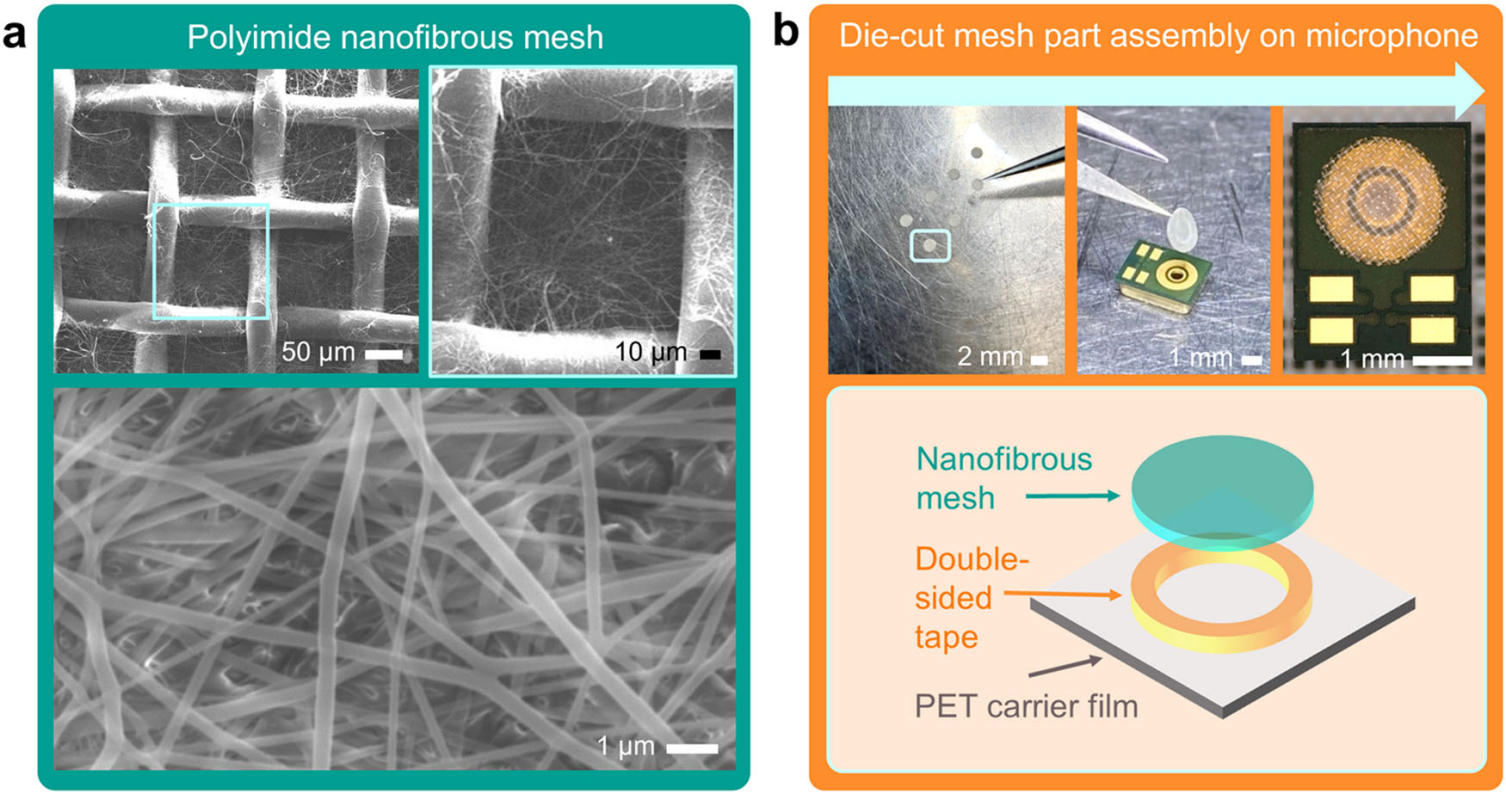
Environmental-protective components combining polyimide (PI) nanofibers onto polyether ether ketone (PEEK)-monofilament-fabric meshes. **a** Scanning electron microscopy (SEM) images of PI-based nanofibrous meshes. **b** Manual assembly and integration process of a die-cut nanofibrous mesh part from a polyethylene terephthalate (PET) liner onto the backside of a packaged microelectromechanical system (MEMS)-based microphone covering its sound port.

To fabricate aromatic polyimide (PI) nanofibers (see Fig. 1a), SAATI SPA used solution-grade P84 powder from Ensinger GmbH (Nufringen, Germany). It is a fully imidized co-PI containing approximately 20% of methyl phenylenediamine and 80% of toluene diamine (BTDA-TDI/MDI). PI was dissolved in N-methyl-2-pyrrolidone (anhydrous 99.5%, NMP) solvent at 20 wt%, in a glass flask using magnetic stirring with a speed of 300 rpm for 2 h at 100 °C. Once the polymer solution had been homogeneous, it was then transferred to an electrospinning process. The electrospinning setup employed a thin stainless-steel wire (0.2 mm) as the spinning electrode and a moving head to apply the polymer solution along the entire length of this wire (see Supplementary Fig. 1). The PI solution was electrospun at room temperature ((22 ± 1) °C) and stable relative humidity ((20 ± 1) %), loaded in the moving head with a stainless-steel orifice of 0.6 mm inner diameter, which was connected to a high-voltage supply to provide a negatively polarized DC voltage of up to 100 kV and a working voltage of 70 kV. The feed rate of the solution was 0.5 rpm, which was controlled by a peristaltic pump. The PEEK-based monofilament fabric mesh with an opening size of 106 µm × 106 µm, an open-area coverage of 56%, a monofilament diameter of 35 μm, and a thickness of 70 µm was fed between the two electrodes as a collector substrate. The fabricated nanofibrous mesh had an air permeability of 1000 l/m^2^/s at 200 Pa, in which during the electrospinning process the substrate speed was set to 0.8 m/min. In the resulting PI nanofibrous meshes, the quantitative content of PI nanofibers is around 33% in weight. The PI nanofibers have average diameters of 200–250 nm (Fig. 1a and Supplementary Fig. 2).

In general, the use of high-density self-standing nanofiber mats results in a nanofibrous mesh having very low air permeability, where the sound transmission occurs through vibration and not via airflow-based mechanism. It is feasible to obtain a self-standing electrospun PI nanofiber membrane alone. However, its high modulus of elasticity, high rigidity, and high degree of hardness can make the resulting materials brittle and difficult to be handled. Consequently, it is desirable to combine the high-temperature (HT) filtering properties deriving from the PI-based nanofibers with the contribution of a HT-resistant carrier (PEEK mesh), an integral part of the membrane, which strengthens the mechanical properties of the final filtering component.

The mechanical properties of the prepared PI nanofibrous mesh were measured using a dynamic mechanical analyzer (DMA 1 STARe System, Mettler Toledo Instruments) in tension mode at a temperature range of 0 – 280 °C and a rate of 5 °C/min, with a constant frequency of 1 Hz. The elastic modulus and stiffness of the nanofibrous mesh were found to be 350 MPa at 25 °C and 18 N/mm, respectively. During measurements, rectangular samples with a dimension of 10 × 8 mm^2^ were employed, in which their thickness was determined via SEM-based cross-section analysis.

After the large-scale hybrid PI-nanofiber-combined PEEK mesh had been prepared in a roll-to-roll process at SAATI SPA, its airborne-particle-filtration tests were performed according to UNI EN ISO 16890. Die-cut parts with an inner diameter of 1.4 mm and an outer diameter of 2.2 mm were produced from it using a flatbed equipment. These die-cut parts have been prepared for coupling one layer of double-sided HT adhesive (Tesa 8854, 100-µm-thick) with SAATI material and sticking it to the neat-woven mesh side. This adhesive material was chosen to allow the entire die-cut parts to sustain the HT reflow cycles without deterioration or deformation. Furthermore, to transfer a die-cut part of SAATI mesh from its carrier of a polyethylene terephthalate (PET) liner onto the printed circuit board (PCB) of a packaged microphone, manual assembly using a tweezer was applied (Fig. 1b). Supplementary Figure 2 depicts a nanofibrous mesh-integrated MEMS microphone in comparison to its bare counterpart.

### Sealed-dual-membrane MEMS microphones

The application of a protective nanofibrous mesh was demonstrated on a digital pulse-density-modulation (PDM) XENSIV^TM^ MEMS microphone (IM72D128V01) from Infineon Technologies AG^12^. The typical sensitivity, A-weighted noise, and SNR values of these microphones in bare conditions without integrated nanofibrous meshes are −36 dBFS, −108 dBFS, and 72 dB, respectively. Furthermore, their low-frequency roll-off (LFRO) and high acoustic overload point (AOP) are at 20 Hz and 128 dBSPL, respectively.

**Fig. 2:**
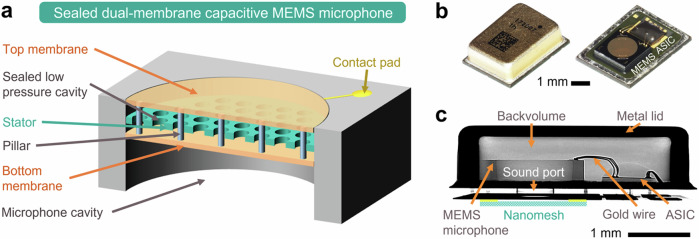
A packaged microphone system comprising a sealed-dual-membrane (SDM) capacitive microelectromechanical system (MEMS), a digital application-specific integrated circuit (ASIC), and a bottom-sound-port package. **a** Three-dimensional (3D) schematic of a capacitive MEMS microphone with SDM technology. Top and bottom membranes are connected by micropillars, in which the air gap inside the cavity has been evacuated resulting in very low pressure. **b** Optical microscopy images of metal-lid-packaged (left) and unpackaged (right) microphone systems. **c** Side-view X-ray image of a packaged microphone. A schematic of a nanofibrous mesh is added below the X-ray image to illustrate its placement above the sound port.

The MEMS microphone chip was developed in a sealed-dual-membrane (SDM) technology^7^, where a highly perforated stator is placed between two polysilicon membranes forming an evacuated cavity (see Fig. 2a). Meanwhile, a digital ASIC applies a constant charge-based differential readout and converts the analog output signal into a PDM bitstream signal via a reconfigurable ΔΣ analog-to-digital converter (ADC). Both MEMS and ASIC chips were attached on a PCB, connected via gold-wire bonding, and packaged inside a metal lid (Fig. 2b, c). The employed microphone package possesses a volume of 4.0 mm × 3.0 mm × 1.2 mm, where its PCB has a circular through hole, the so-called sound port, with a diameter of 0.6 mm, which is placed underneath the MEMS chip. Furthermore, a die-cut part of nanofibrous mesh was placed outside the package covering the sound port during acoustic measurement and robustness evaluation (Figs. 1b and 2c).

## Results and discussion

### Acoustic impedance of nanofibrous mesh

**Fig. 3:**
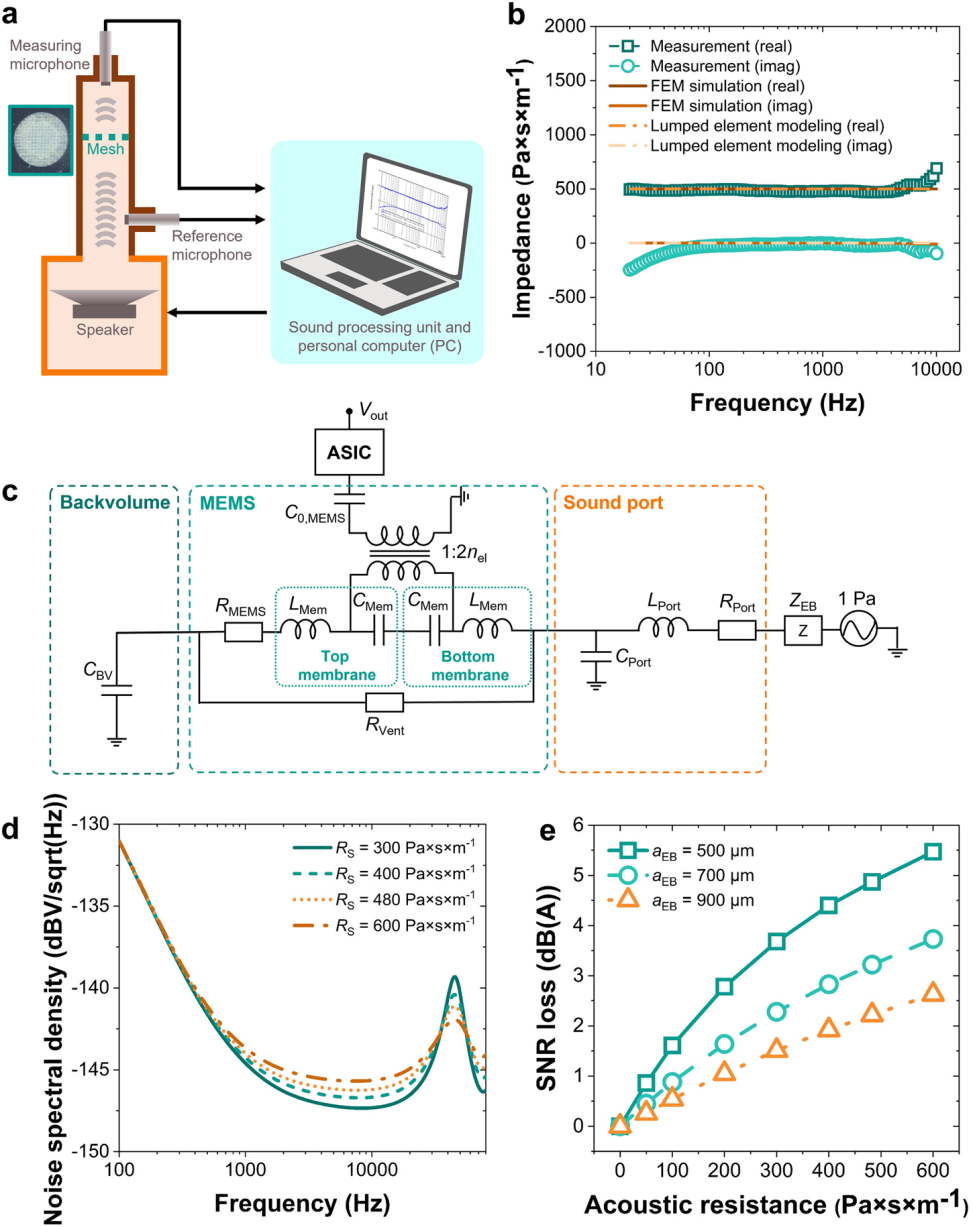
Acoustic effects of nanofibrous mesh on microelectromechanical system (MEMS)-based microphone performance. **a** Test setup for measuring acoustic impedance of nanofibrous mesh. **b** Typical real and imaginary (imag) acoustic impedances of a die-cut nanofibrous mesh part with an inner diameter of 1.4 mm at different frequencies obtained from measurement, finite element method (FEM) simulation, and lumped element modeling. **c** Equivalent-circuit model of a mesh-protected microphone, comprising elements of MEMS, application-specific integrated circuit (ASIC), package, and nanofibrous mesh. **d** Impact of the specific acoustic resistance *R*_s_ of a nanofiber mesh with die-cut-part radius *a*_EB_ = 700 µm on the output noise of a capacitive MEMS microphone. **e** Simulated signal-to-noise ratio (SNR) loss of capacitive MEMS microphones protected by nanofibrous meshes of various specific acoustic resistances *R*_EB_ and radii *a*_EB_.

The acoustic properties of the nanofibrous mesh die-cut parts were studied by measuring their acoustic impedances using a custom impedance tube (MAE103c, SAATI SPA). This setup was specifically designed to measure the complex acoustic impedance of samples having small areas in the millimeter range and high-magnitude impedances (see Fig. 3a). The acoustic-impedance characterization system comprises a drive speaker, a vibration-damping transmission tube, a sample holder with both measurement and reference microphones, and a microphone amplifier. The system works by driving the speaker, which sends an acoustical wave up through the tube. The signal is usually a sequence of pure tones with increasing frequency that generates a standing wave condition in the tube. The wave passes through the sample (i.e., the nanofibrous mesh) mounted in the sample holder above the measuring microphone to the reference microphone (free, not covered by the sample). A commercial ARES acoustic software built by SAATI SPA^30^ is used to process the microphone signals and subsequently determine the pressure drop and velocity through the sample, from which the acoustic impedance is calculated. Here, the acoustic impedance is the ratio of pressure drop across an acoustical part divided by the volume velocity through it. This quantity is not a single value, instead comprises parameters of a wave signal. Thus, complex phasors are used by the measurement system to display both magnitude and phase or real and imaginary parts, respectively, of the acoustic impedance curve with respect to frequency. Sound cards with a proper calibration are used to generate the speaker signal and measure the microphone response. Moreover, specific procedures are also typically required for calibrating a microphone and its amplifier. A sample holder can be used to place the parts with inner diameter between 1 – 3 mm (free area of the sample), while the total outer dimension of the die-cut part must be <10 mm. The tube with a size of ~25–30 cm can be placed on a desk next to the personal computer (PC).

The measured complex-valued specific-acoustic-impedance curves of a die-cut nanofibrous mesh part are displayed in Fig. 3b, which are composed of real and imaginary parts. At 1 kHz, the real and imaginary (imag) acoustic impedances of this mesh are 479.99 Pa×s×m^−1^ and −4.47 Pa×s×m^−1^, respectively. The measured specific acoustic impedance of the mesh is therefore strongly dominated by its real part accounting for dissipation effects.

Porous acoustic materials like the nanofibrous SAATI meshes described in this study can generally transmit sound waves in two possible ways, i.e., by the airflow passing through the pores of the material and/or the vibration of the material itself. While airflow through its pores leads to viscous dissipation and therefore primarily affects the real part of the acoustic impedance, vibrational effects such as spring-stiffness and inertia are dominating the imaginary part of impedance. The effect of a porous material in an acoustic circuit can therefore generally be modeled as two acoustic impedances in parallel. While the first impedance path corresponds to the specific airflow resistance through the pores of the material, the second impedance path represents vibrational contributions, which are influenced by the mechanical properties of the bulk material and/or its support^31^. Our die-cut parts allow to combine large airflow passage and high stiffness provided by the supporting PEEK mesh.

Besides acoustic-impedance-measurements, we also performed finite element method (FEM) simulation using the ARES software^30^ and lumped-element modeling described in the next section to confirm the resulting measured acoustic impedances and to better design the protective materials in acoustic circuits^32–34^. The ARES acoustic tool contains a dedicated FEM module representing an exhaustive behavior description of the tested material, which includes both the flow-through and vibrational paths.

From the results shown in Fig. 3b, the die-cut nanofibrous mesh part with an inner diameter of 1.4 mm has negligible vibrational contribution to the sound transmission, which is indicated by the FEM-simulated and lumped-element-modeled imaginary acoustic impedances of −0.94 Pa × s × m^−1^ and 0 Pa × s × m^−1^, respectively. This has validated the high stability and stiffness of the PEEK mesh, which is used to support the deposited PI nanofibrous mats. In other words, the specific acoustic impedance of a nanofibrous-mesh die-cut part is almost completely composed of the resistance of the flow-through path, which has neither a frequency dependency nor an imaginary part^35^. All in all, it is evident that there is a good agreement of results obtained from the three described investigation methods (i.e., measurement, ARES-based FEM simulation, and simple lumped modeling). Minor deviations only start appearing at very high frequencies (around 10 kHz), which are mainly caused by the limitation of the instrumentation tools. The outputs of these acoustic impedance simulations and measurements were then used in the system simulation of the complete device.

### Lumped-element-based modeling of nanofibrous mesh-integrated MEMS microphones

The impact of the nanofibrous mesh acoustic impedance on the microphone performance was theoretically investigated via a lumped-element-based equivalent circuit model, considering the MEMS, ASIC, and package. Lumped-modeling-based approaches have been conventionally applied to simulate capacitive MEMS microphones, particularly focusing on their sensitivity, noise, and SNR^36,37^. Due to their inherited order reduction to a single-dimensional equivalent circuit representation, the developed models allow for very fast and computationally effective simulations. A lumped element model representation of the nanofibrous-mesh-protected microphone is depicted in Fig. 3c. The MEMS comprises two stiffly coupled circular membranes (top and bottom membranes) with a radius of *a* and a thickness of *t*_m_. Finding formulations for their respective mass and spring constant requires to account for flux and energy conservation in the reduced-order lumped representation. Assuming the membranes to be under high tensile stress allows to approximate their deflection shape in dependence on the radial position $\vec{r}$ from the center due to incident quasi-stationary pressure by

1 $$z\left(\vec{r}\right)={z}_{{pk}}\left(1-\frac{{\vec{r}}^{2}}{{a}^{2}}\right)=2{z}_{{avg}}\left(1-\frac{{\vec{r}}^{2}}{{a}^{2}}\right)$$

where *z*_pk_ corresponds to the membrane deflection amplitude in the center and *z*_avg_ is the area-averaged deflection. The kinetic mass *m* of the membrane can be estimated by ensuring a conservation of its kinetic energy in both distributed (Eq. (2)) and lumped (Eq. (3)) formulations:

2 $$\frac{1}{2}m{\left(\omega {z}_{{avg}}\right)}^{2}=\frac{1}{2}{\int }_{\!\!\!\!0}^{a}2\pi {t}_{m}\rho \, {\left[\omega z\left(\vec{r}\right)\right]}^{2}\vec{r}\,d\vec{r} ,$$

3 $$m=\frac{4}{3}\rho {\pi {t}_{m}a}^{2}.$$

Where *ω* and *ρ* are the angular frequency and the density, respectively. The acoustic lumped mass ${L}_{{mem}}$ in Fig. 3c then corresponds to

4 $${L}_{{mem}}=\frac{\frac{4}{3}\rho {\pi a}^{2}}{({{a}^{2}\pi })^{2}}=\frac{4\rho }{3{\pi a}^{2}}.$$

The mechanical spring constant *k* of the membrane can be extracted from its vacuum resonance frequency *f*_0_ by

5 $${f}_{0}=\frac{1}{2\pi }\sqrt{\frac{k}{m}}.$$

The acoustic membrane compliance *C*_mem_ of the membrane results to

6 $${C}_{{mem}}=\frac{{\left({a}^{2}\pi \right)}^{2}}{k}=\frac{{\left({a}^{2}\pi \right)}^{2}\,}{m{\left(2\pi {f}_{0}\right)}^{2}}\,.$$

Oscillations of the membrane further alter the gap separating it from the counter electrode and cause capacitance changes. Charging the MEMS capacitance *C*_0,MEMS_ via a bias voltage *V*_bias_ converts the capacitance changes into an output voltage *V*_out._ Assuming a linearized working point, the described electro-mechanical coupling of a single membrane and the counter electrode can be approximated via an ideal transformer with a coupling factor of *n*_el_:

7 $${n}_{{el}}=\frac{{a}^{2}\pi {V}_{{bias}}}{m{\left(2\pi {f}_{0}\right)}^{2}{t}_{0}}.$$

Movement of the two coupled membranes with respect to the counter electrode causes its two sub-capacitances to change out-of-phase. Subtracting the two corresponding signals by applying a differential readout therefore doubles the output voltage of the microphone and allows both airgaps to be described by a single ideal transformer of coupling factor *n*_el,diff_ = 2*n*_el_.

The MEMS counter electrode is mechanically approximated to be ideally rigid and described by a purely resistive circuit component *R*_MEMS_ accounting for viscous dissipation occurring within its perforation holes. Airflow through the circular venthole in the membranes is designated by a resistance *R*_Vent_^36^. For a description of the full microphone module, the MEMS model needs to be further coupled to descriptions of the package. The chip cavity of the MEMS and sound port in the underlying PCB form a Helmholtz-resonator-shaped geometry, which can be represented by a linear harmonic oscillator with three circuit components (*R*_Port_, *L*_Port_, and *C*_Port_). Expressions for the corresponding lumped components depend on the resonator geometry^38^. The metal lid covering the membrane and the ASIC further encloses an air volume, the so-called backvolume (Fig. 2c). Membrane oscillations compress and expand this volume, leading to a spring-like counter-force acting on the membrane described by a lumped acoustic capacitance *C*_BV_^39^.

The microphone-lumped model is finalized by enclosing the sound port with the acoustic impedance *Z*_EB_ of the nanofibrous mesh. Figure 3b shows the specific acoustic impedance of the nanofibrous mesh to be dominated by its real part and therefore allows to approximate its impedance by an acoustic resistance *R*_EB_:

8 $${R}_{{EB}}=\frac{{R}_{s}}{{a}_{{EB}}^{2}\pi },$$

where *R*_s_ = R_e_(Z_s_) corresponds to the real part of its specific acoustic impedance and *a*_EB_ is the mesh radius. The pressure noise *P*_n_ generated by an acoustical resistance *R*_EB_ can be analytically formulated to

9 $${P}_{n}=\sqrt{4{k}_{b}T{R}_{{EB}}}=\sqrt{4{k}_{b}T\frac{{R}_{s}}{{a}_{{EB}}^{2}\pi }} ,$$

where *k*_b_ and *T* are the Boltzmann constant and the ambient temperature, respectively. A low-pressure noise *P*_n_ and therefore a low impact on the microphone’s signal-to-noise ratio can be achieved via either a low specific acoustic mesh resistance *R*_s_ or a large mesh area ${a}_{{EB}}^{2}\pi$.

The lumped-element-based simulation result in Fig. 3d shows the impact of a varying real part of the specific acoustic impedance *R*_s_ with radius *a*_EB_ = 700 µm on the electrical output-noise spectral density of the nanofibrous-mesh-protected microphone. In accordance with Eq. (9), an increasing specific acoustic resistance *R*_s_ enlarges the output noise of the microphone and should therefore impair its SNR. The effects of changing specific acoustic resistance *R*_s_ and mesh radius *a*_EB_ on the simulated SNR are depicted in Fig. 3e. Based on Eq. (9), both increasing *R*_s_ and reducing radius *a*_EB_ lead to a higher thermal noise and thereby enlarge the SNR loss of the microphone. From the simulation, considering the nanofibrous mesh characterized in Fig. 3b with *R*_s_ = 480 Pa × s × m^−1^ and *a*_EB_ = 700 µm, an SNR loss of 3.2 dB(A) can be estimated. Note that these losses do not originate from wave reflection due to an impedance mismatch of the nanofibrous mesh with regards to the environment. Instead, they arise solely from thermal noise generated by viscous dissipation. The detailed parameters and their values used in lumped-element-based simulation are listed in Supplementary Table 1.

### Electroacoustic characterization of nanofibrous mesh-integrated microphones

After performing the simulation to estimate the SNR loss of the nanofibrous-mesh-combined MEMS microphone system, electroacoustic measurements of the fabricated MEMS microphones were conducted in an anechoic free-field chamber provided by Infineon Technologies AG, which comprises a loudspeaker and a reference microphone (see Supplementary Fig. 3). The free-field chamber is a foam-filled cubic enclosure that is designed to enable a disturbance-free propagation of acoustic waves, mimicking an acoustic free-field. The foam filling can prevent acoustic reflection from the chamber wall and suppress geometrical resonance along its inner dimension. Moreover, the metal housing and foam-filling of the chamber can reflect and absorb external acoustic noise, respectively, and thereby acoustically shield the measurement setup from the environment. During acoustic sensitivity measurement, both MEMS microphone and reference microphone were placed in ~50 cm distance from the loudspeaker. The MEMS microphone sensitivity was determined by monitoring its output voltage at 1 Pa of sound pressure level (SPL) produced by the loudspeaker. The loudspeaker was equalized to 1 Pa within the audible frequency range of 20 Hz to 20 kHz via a reference microphone. The monitoring of the output voltages from the MEMS microphone as a device under test (DUT) and reference microphone and the actuation of the loudspeaker were performed by an audio analyzer that was connected to a personal computer (PC).

**Fig. 4:**
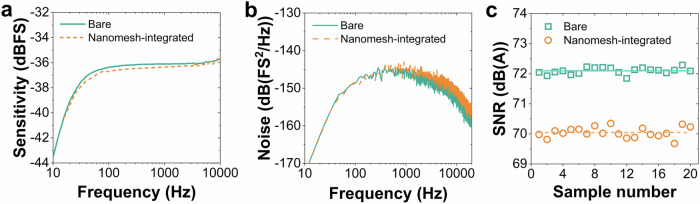
Electroacoustic performance of nanofibrous mesh-integrated microelectromechanical system (MEMS) microphones. Electroacoustic measurement results showing **a** sensitivity values at frequencies from 10 Hz to 10 kHz, **b** A-weighted noise spectral densities at frequencies from 11 Hz to 20 kHz, and **c** measured signal-to-noise ratio (SNR) values of bare and nanofibrous mesh-protected MEMS microphones.

Here, the electroacoustic characteristics of microphone samples before and after integrating the nanofibrous meshes (see Supplementary Fig. 2a) were measured and subsequently compared to deduce insertion and SNR losses. To have a quantitative analysis, 20 samples were characterized, where the average and standard deviation of the measured data were calculated to verify reproducibility. From the frequency responses in Fig. 4a, it is obvious that both bare and nanofibrous mesh-integrated microphones demonstrate similar sensitivity- curve characteristics where a flat response trend is obtained in the audible frequency range. Moreover, measuring the MEMS microphone output voltage without speaker actuation results in the microphone noise, in which its A-weighted value can be combined with that of sensitivity to determine SNR.

Table 1 lists all key performance indicators (i.e., sensitivity, A-weighted noise, and SNR) of the MEMS microphones before (bare) and after nanofibrous-mesh integration. In accordance with the theoretical simulation approaches, the nanofibrous mesh causes thermal noise and thus primarily increases the microphone noise spectral density (see Fig. 4b). The measured A-weighted noise of nanofibrous mesh-integrated microphones is (−106.48 ± 0.16) dBFS. This value is (1.75 ± 0.15) dBFS higher than that of bare microphones. Moreover, the lack of vibrational contribution of the nanofiber material on the SNR loss has also been confirmed, where the microphone sensitivity is only slightly altered after integrating the mesh component resulting in an insertion loss of (0.30 ± 0.11) dBFS. Considering those two affecting components (i.e., noise increase and insertion loss) on the system, a measured SNR loss of (2.05 ± 0.16) dB(A) can be determined. Despite having this acoustic effect, the nanofibrous mesh-integrated microphone still can maintain a high SNR value of (70.05 ± 0.17) dB(A) as depicted in Fig. 4c, which is beneficial to obtain high-quality sound-capturing signals during audio recordings.

**Table 1:** Electroacoustic performance comparison of bare and nanofibrous mesh-integrated microelectromechanical system (MEMS) microphones

Microphone type	Sensitivity (dBFS)	A-weighted noise (dBFS)	SNR (dB(A))
Bare microphones	(−36.14 ± 0.11)	(−108.23 ± 0.07)	(72.10 ± 0.11)
Nanofibrous mesh-integrated microphones	(−36.44 ± 0.11)	(−106.48 ± 0.16)	(70.05 ± 0.17)

Sensitivity, noise, and signal-to-noise ratio (SNR) are used as key performance indicators for both microphone types.

### Robustness tests of die-cut nanofibrous mesh parts

**Fig. 5:**
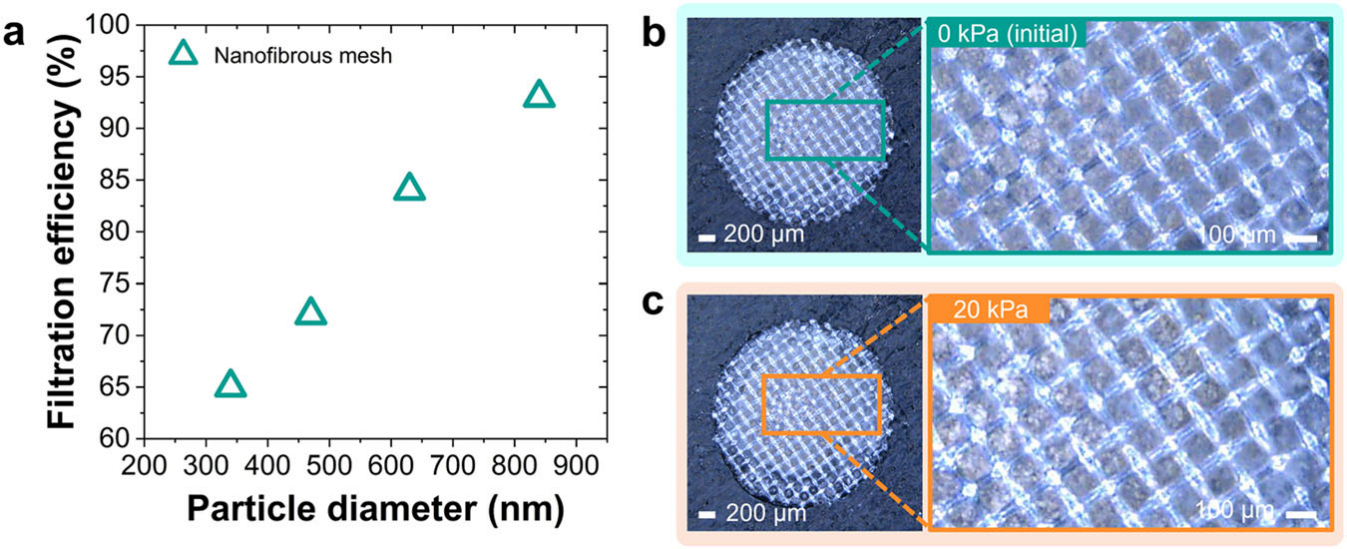
Robustness tests of die-cut nanofibrous mesh parts. **a** Filtration efficiency of nanofibrous meshes tested for particles with different sizes. Optical microscopy images of a nanofibrous mesh (**b**) before and (**c**) after being applied with a static pressure of 20 kPa. The PI-based nanofibrous structure appears to be unaltered, proving the stability of the nanomesh part not yet installed on the microelectromechanical system (MEMS) during processing conditions.

In addition to electroacoustic performance characterization, the developed nanofibrous meshes were also investigated in two different robustness tests. First, an airborne particle filtration test was conducted as an environmental robustness evaluation to confirm the capability of the die-cut nanofibrous mesh part to trap the flowing particles approaching to microphone (see Fig. 5a). The particle filtration test was performed according to the international standard ISO 16890, which has replaced the previous standard in Europe for testing air filters (i.e., EN 779) in July 2018^40^. ISO 16890 has adopted a classification method following the recommendation from the world health organization (WHO) on particulate matter (PM) size^41^. Unlike the previous and outdated EN 779 standard, which used 0.4 μm particles to measure the efficiency of filters, the ISO 16890 standard tests the particle- filter performance (filtration efficiency) for different particle sizes (i.e., PM_1_, PM_2.5_, and PM_10_). PM_1_ represents particles with a diameter of less than or equal to 1 µm (ultrafine particles or nanoparticles). PM_2.5_ corresponds to particles with a diameter of less than or equal to 2.5 µm (fine particles). PM_10_ belongs to particles with a diameter of ≤10 µm. Furthermore, two types of test aerosols were used in this standard test (i.e., a liquid-phase aerosol of di-ethyl-hexyl-sebacate (DEHS) and polydisperse solid-phase potassium chloride (KCl) generated from aqueous solution). The DEHS and KCl aerosols could generate particles with diameter-size ranges of 0.3 µm–1.0 µm and 1.0 µm–10.0 µm, respectively.

Here, particles with four different sizes (i.e., 340, 470, 630, and 840 nm) were employed for analysis. To determine the particle-filtration efficiency, the number concentrations of flowing particles before and after passing through the die-cut mesh part were compared. The larger the tested particles, the higher the filtration efficiency obtained by the mesh. The nanofibrous mesh having a quantitative content of PI nanofibers of ~33% in weight has reached its highest particle-filtration efficiency of 93% when it is tested to filter out 840-nm-large particles, as shown in Fig. 5a. The resulting nanofibrous mesh was classified as air filter PM_1_ for particles with sizes of <1 μm and >0.3 μm.

Decreasing nanofiber density would lead to a worsening of filtration efficiency. This impact has been proven from the case of mesh sample having a lower quantitative content of PI nanofibers of ~25% in weight, which has yielded a lower particle filtration efficiency of 82% when tested to filter 840-nm-large particles. Thus, to reach the target of higher particle-filtration efficiency, higher density of nanofibers is preferable to be integrated onto the mesh.

It is understood that a trade-off exists for the correlation between acoustic performance (SNR loss) and environmental robustness (IP level) towards solid particles for such protective mesh structures. Meshes with denser and thicker deposited nanofibers are expected to possess a higher IP level towards particles, but simultaneously suffer from a higher SNR loss in the microphone system. Thus, the PI nanofiber density deposited on the PEEK mesh has been carefully set according to the target microphone applications. Again, for this experiment, since the employed bare packaged microphones have already had a high intrinsic protection level towards dust and water (IP57) due to their robust SDM architecture^12^, relatively loose nanofibers with low thickness are sufficient in order not to deteriorate the acoustic performance heavily. Another strategy to avoid high SNR loss is simply by enlarging the inner diameter of the die-cut mesh part that can reduce the specific acoustic resistance (Fig. 3e).

The second investigation is a pressure-step test, typically used to characterize the mechanical robustness of membranes withstanding high pressure, which was conducted on the die-cut nanofibrous mesh part in this case. In a static pressure test setup, a 3D-printed nozzle was used to apply pressure and was positioned on top of a tested sample, creating a tight air sealing. The nozzle inner diameter was set to be slightly larger than that of the mesh die-cut part. Hence, no direct contact would occur at the active and touch-sensitive part of the sample. The regulation stated a gradual increase of the pressure from zero to the defined setpoint, where optical inspection was performed after each step. As the regulation requires infinite back volume (different from the real case of the MEMS microphone), the die-cut nanofibrous mesh part was first mounted on a mechanical support. Hence, a gap between the tested mesh and its underneath metal plate was realized.

Here, it is possible to test the die-cut nanofibrous mesh part on applied pressure levels up to 800 kPa. However, in this case, a meaningful value of pressure could be 20 kPa (Fig. 5b, c and Supplementary Fig. 4). This condition corresponds to an acoustic pressure of 180 dBSPL, which is already beyond the typical maximum sound pressure level encountered in consumer applications (e.g., smartphones). Therefore, for the tested nanomesh-integrated microphones, the interesting pressure level extends up to 20 kPa. As visible in Fig. 5c, at 20 kPa the nanofibers retained their integrity. For completeness, the pressure had been increased up to 50 kPa, that is the upper limit for the tested part, at which the overlaid nanofibers started breaking in few regions of the mesh.

### Environmental robustness tests of nanofibrous mesh-integrated MEMS microphones

**Fig. 6:**
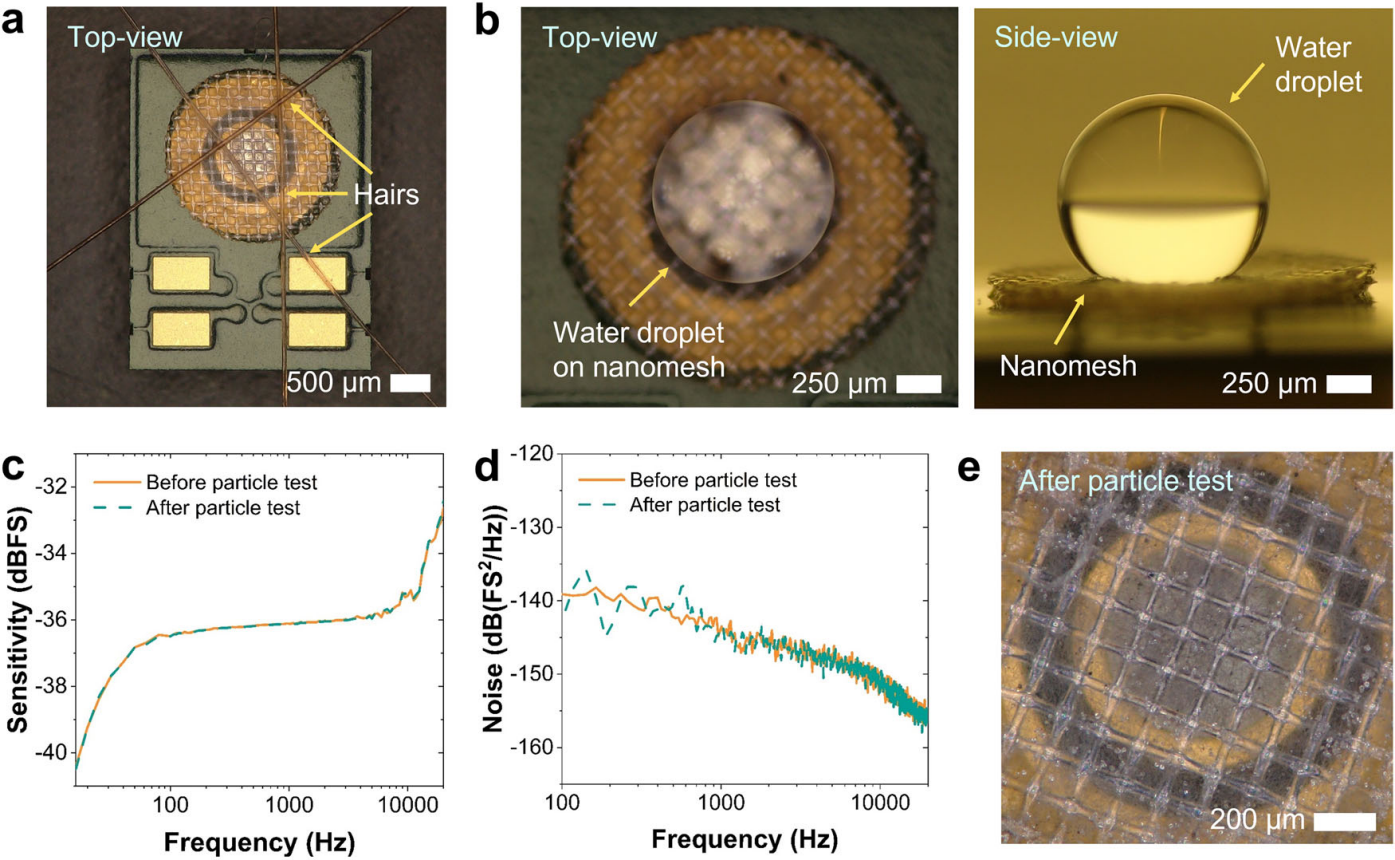
Environmental-robustness-test results of nanofibrous mesh-integrated microelectromechanical system (MEMS) microphones. **a** Hair-drop test depicting the capability of a nanofibrous mesh to protect the microphone sound port from hairs. **b** Water contact angle (WCA) measurement exhibiting high surface hydrophobicity of the nanofibrous mesh. Images were captured from the top (left) and the side (right). **c** Typical sensitivity, (**d**) A-weighted noise spectral density, and (**e**) optical microscopy image of a nanofibrous mesh-integrated MEMS microphone after dust-particle test.

Besides robustness tests at the component level (i.e., only nanofibrous mesh), further environmental robustness evaluation was performed at the system level (i.e., with completed nanofibrous mesh-integrated MEMS microphones). These include hair drop, surface wettability, and dust microparticle tests, where their results are displayed in Figs. 6a–e and Supplementary Figs. 5–7. First, a qualitative hair drop test was carried out to investigate the ability of the nanofibrous mesh to shield the MEMS microphones from hairs for the case of their applications in consumer electronics (e.g., headphones, smartphones, or true wireless earbuds/earphones). The fiber properties (shape, size, curl, and color) of human hairs can vary across different countries^42^, where their typical diameters are in the range of 60–200 µm. Here, we picked three human hairs with a light brown color using a tweezer and dropped them onto a nanofibrous mesh-integrated microphone from a distance of ~0.5 cm. From Fig. 6a and Supplementary Fig. 5, the hairs can be filtered out and do not penetrate to the microphone sound port. Since hairs have an elongated shape (i.e., their longitudinal dimension (length) is much larger than their transversal one (cross-sectional diameter)), they could by-pass the shielding mesh only if they are perpendicularly aligned to the mesh surface and have sufficient force to break the nanofibers. Nonetheless, the possibility of such this situation to occur is rather low, or even close to zero for the case of long hairs^11^.

Second, surface wettability tests in the form of water contact angle (WCA) measurements were conducted on the nanofibrous meshes that had been attached onto the backside of MEMS microphones to investigate their characteristics towards water droplets. An average WCA value of (132.8 ± 5.1)° was obtained from five measured nanomesh-on-microphone samples (Fig. 6b and Supplementary Fig. 6). These results have demonstrated high surface hydrophobicity of the nanofibrous meshes due to the high intrinsic hydrophobicity of PI^28,29^. Hence, water droplets coming from a splash can be trapped on the nanomesh surface and do not enter the sound port. To further enhance their surface hydrophobicity, the already electrospun nanofibrous meshes can be optionally treated in plasma-enabled hydrophobic polymer coating process in the production.

Lastly, since the microphones have normally been exposed to dust particles from environment during their use in real applications, we also carried out dust microparticle tests for the built nanofibrous mesh-integrated MEMS microphones as well as evaluated their electroacoustic properties and surface conditions (Figs. 6c–e and Supplementary Figs. 7a–e). Here, polydisperse native starch particles having a size distribution of 0.9–61.0 µm were employed as test dusts. Such particle types were also used in aerosol-related medical research^43^. During the experiment inside a sealed tube with a diameter of ~10 cm and a height of ~30 cm, the mesh-integrated microphone samples were positioned next to a small bowl filled up with dust particles. The particle exposure to samples occurred when the air inside the tube was blown resulting in deposition of particles from the air onto the backside surfaces of microphones (see Supplementary Fig. 7). After being exposed with starch microparticles, the samples were again measured in terms of their electroacoustic characteristics. Here, the sensitivity of the samples remained almost unaltered (Fig. 6c and Supplementary Fig. 7a). A slight noise increase of (0.11 ± 0.11) dBFS was found (Fig. 6d and Supplementary Fig. 7b), which was caused by the particles deposited on the nanofibrous mesh. These trapped particles shown in Fig. 6e and Supplementary Fig. 7e induce additional acoustic resistances because they partially block the pathways for the airflow passing through the pores in the nanomesh, which then results in higher noise. Consequently, an average SNR loss of (0.14 ± 0.13) dB(A) was notably observed (Supplementary Fig. 7c**)**.

In case the trapped particles have excessive number, high packing density, and strong adhesion to the mesh, they may completely block several pores of the nanofibrous mesh preventing the airflow to pass through them. This can result in a performance degradation of the microphone. Thus, strategies to detach the already deposited particles from the mesh surface are required to be developed. In other studies, several particle removal techniques have been proposed, including ultrasonic cleaning for ceramic membrane^44,45^ and silicon nanowire resonators^46,47^, filter shaking^48,49^, reverse pulsed-flow cleaning^50^, silicone molding^51^, and chemical cleaning^52^. Among them, ultrasonic cleaning and device shaking can be potential candidates for the regeneration of nanofibrous mesh-integrated MEMS microphones that have been fully loaded with particles having low adhesion forces to the mesh surfaces.

In comparison to the glass micromesh chip produced by laser-induced deep etching (LIDE)^11^, the die-cut nanofibrous mesh part presented in this study demonstrate its superiority in terms of particle-filtering capability. The nanofibrous mesh can filter nanoparticles with sizes of <1 µm, while the glass mesh is only able to filter large microparticles with sizes of >80 µm. This feature has been enabled by the unique combination of electrospun PI nanofibers with a PEEK mesh. However, this advantage of having porous structures and smaller mesh opening sizes (<1 µm) also comes with larger penalty in electroacoustic performance. Using the same SDM-MEMS microphones as a test vehicle, the SNR loss yielded by nanofibrous mesh (i.e., (2.05 ± 0.16) dB(A)) is around three times higher than that of glass mesh (i.e., (0.65 ± 0.05) dB(A)). Nonetheless, this SNR loss can be adjusted or reduced by changing the electrospinning process parameters, which subsequently results in altered nanofiber properties (e.g., lower nanofiber density or larger mesh-opening size can yield higher SNR). A detailed comparison between the nanofibrous mesh and its glass counterpart is listed in Table 2.

**Table 2:** Comparison of meshes used as protective components for microelectromechanical system (MEMS)-based microphones

Parameter	Glass mesh	Nanofibrous mesh
Material	Glass	PI nanofibers combined onto PEEK fabric
Fabrication method	Laser-induced deep etching (LIDE)	Electrospinning
Scalability	8-inch-wafer-level scale	Large scale (roll-to-roll)
Compatibility with semiconductor industry processing	High	Low
Die shape and size	Rectangular; chip size = 1.7 × 1.7 mm^2^	Circular; inner diameter = 1.4 mm, outer diameter = 2.2 mm
Mesh opening size	80 µm	<1 µm
Employed MEMS microphone	SDM-MEMS microphone	SDM-MEMS microphone
Sensitivity change	(0.11 ± 0.04) dBFS	(0.30 ± 0.11) dBFS
SNR loss	(0.65 ± 0.05) dB(A)	(2.05 ± 0.16) dB(A)
SNR of mesh-protected MEMS microphone	(71.24 ± 0.11) dB(A)	(70.05 ± 0.17) dB(A)
Particle filtering capability	Microparticles ( >80 µm)	Nanoparticles ( <1 µm)
Water contact angle	(133.1 ± 10.3)°	(132.8 ± 5.1)°
Reference	Acanfora et al. (2023)^11^	This work

Two different meshes (i.e., nanofibrous mesh and glass mesh) are compared mainly in terms of their materials, fabrication methods, acoustic performances, and environmental robustness levels.

Based on these results, PI nanofibrous meshes on the one hand allow to protect MEMS microphones from micro- and nanoparticles and on the other hand only have minor impact on their acoustic performance. For the future work, several strategies can be proposed to improve the mechanical properties of the electrospun PI nanofibers, e.g., higher temperature imidization^53^, direct nanoscale-filler dispersion^54^, homogeneity reinforcement^55^, and composite nanofiber creation^56^.

Depending on several factors (e.g., MEMS chip type, package concept, and number of ordered microphones), the costs of packaged MEMS microphones are in the range of 0.25 USD for low-end microphones up to more than 1 USD for specialized devices in demanding markets and applications. All the considered impacts of the protective component materials will not substantially influence the overall costs and will be sustainable with the improvement requirements of device performances.

## Conclusions

Environmental protective components combining polyimide (PI) nanofibers with polyether ether ketone (PEEK) monofilament fabric meshes have been developed for microelectromechanical system (MEMS)-based acoustic sensing applications. Their main electroacoustic parameters (sensitivity, noise, and signal-to-noise ratio (SNR)) have been evaluated theoretically and experimentally using high-performance SDM-MEMS microphones in an industry-standard setup. The fabricated nanofibrous mesh die-cut parts with inner diameter of 1.4 mm offer low insertion and SNR losses of (0.30 ± 0.11) dBFS and (2.05 ± 0.16) dB(A), respectively, when being integrated onto packaged microphones. Thus, the SNR of nanofibrous mesh-integrated microphones can be maintained at a high value of (70.05 ± 0.17) dB(A). A submicron particle filtration efficiency of up to 93% has been obtained by the nanofibrous mesh proving its effective functionality as an environmental protective element. Based on the results from system-level robustness tests, the developed protective components are proven not only being able to enhance the environmental protection level of MEMS microphones, but also possessing only minor adverse impact on their acoustic performance.

## Supplementary information

Supplementary Information

## Data Availability

All data supporting the findings of this study are available within the article and Supplementary Information files. They are also available from the corresponding authors upon reasonable request.

## Code availability

The codes used in this study are available from the corresponding authors upon reasonable request.
