# Supplementary material for: Acoustically semitransparent nanofibrous meshes appraised by high signal-to-noise-ratio MEMS microphones
Source: Commun Eng. 2024 Sep 23;3:136. doi: 10.1038/s44172-024-00283-4 (PMC11420236; doi:10.1038/s44172-024-00283-4)
Supplement: Supplementary file 1 — Supplementary Information [file 44172_2024_283_MOESM1_ESM.pdf]

Supplementary Information

Acoustically semitransparent nanofibrous meshes appraised by high signal-to-noise-ratio MEMS microphones

Hutomo Suryo Wasisto[1,*], Sebastian Anzinger[1], Giovanni Acanfora[1,2], Aloysius Farrel[1,3], Valentina Sabatini[4], Elisa Grimoldi[4], Vasco Marelli[4], Nikita Ovsiannikov[1,5], Konstantin Tkachuk[1], Giordano Tosolini[1], Carmine Lucignano[4], Marco Mietta[4], Guangzhao Zhang[1], Marc Fueldner[1], and Erwin Peiner[2,*]

[1] Infineon Technologies AG, Am Campeon 1-15, 85579 Neubiberg, Germany
[2] Institute of Semiconductor Technology (IHT) and Laboratory for Emerging Nanometrology (LENA), Technische Universität Braunschweig, Hans-Sommer-Str. 66, 38106 Braunschweig, Germany
[3] TUM School of Natural Sciences, Technische Universität München, James-Franck-Str. 1, 85748 Garching, Germany
[4] SAATI SPA, Via Milano 14, 22070 Appiano Gentile, Italy
[5] TUM School of Computation, Information, and Technology (CIT), Technische Universität München, Arcisstraße 21, 80333 München, Germany

[*] Corresponding authors. E-mails: hutomosuryo.wasisto@infineon.com (H.S.W.); e.peiner@tu-braunschweig.de (E.P.)

Supplementary Notes

Supplementary Note 1 .... S-2
Supplementary Note 2 .... S-3
Supplementary Note 3 .... S-4
Supplementary Note 4 .... S-5
Supplementary Note 5 .... S-6
Supplementary Note 6 .... S-7
Supplementary Note 7 .... S-8
Supplementary Note 8 .... S-9

Supplementary Notes

Supplementary Note 1

Electrospinning setup

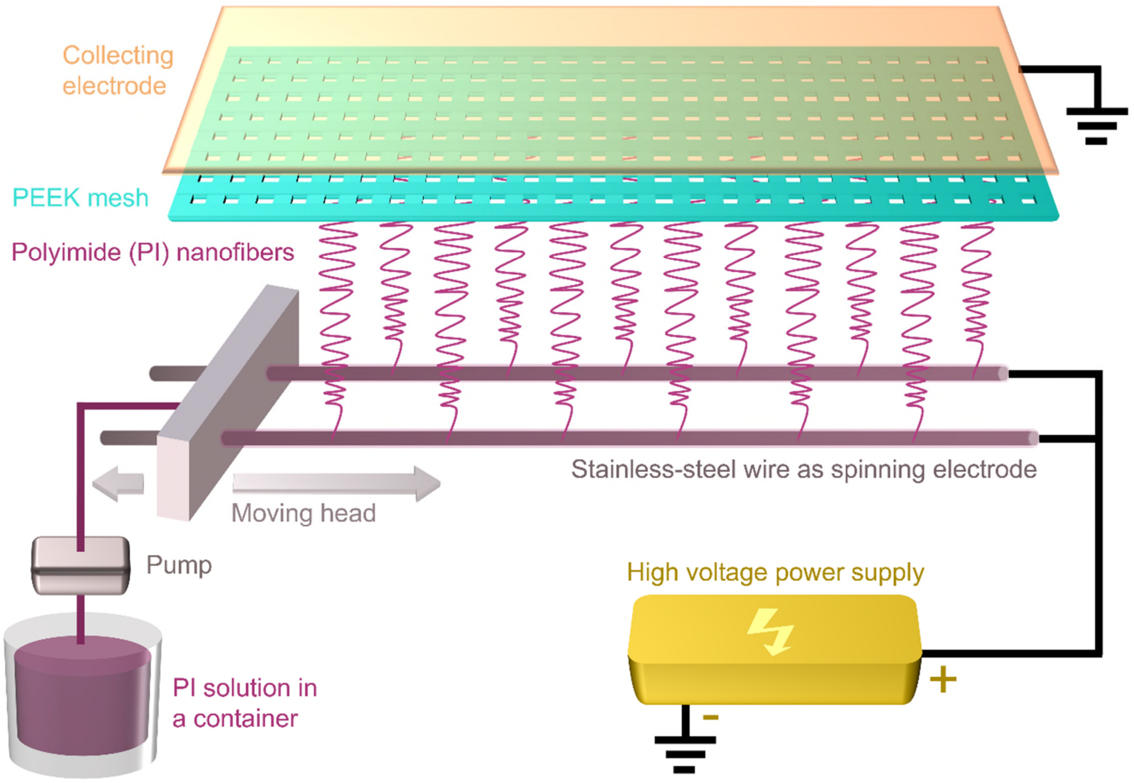

Supplementary Figure 1. Industrial-scale electrospinning setup to fabricate polyimide (PI) nanofiber-combined PEEK mesh. The electrospinning setup mainly comprises thin stainless-steel wires as a spinning electrode, a moving head for applying the PI solution along the entire length of these wires, and a metal plate as a collecting electrode. PI nanofibers are formed from the thin PI layer on the spinning electrode by applying high electrical field using high voltage power supply. These PI nanofibers are collected on polyether ether ketone (PEEK) mesh that is located under the collecting electrode. The used PEEK mesh is prepared in a roll-to-roll fashion. Here, only a small amount of PI solution is applied to the spinning wire electrode during each cycle of the moving head, which is a prerequisite for yielding consistent morphology and homogenous deposition of nanofibers during long-term production.

Supplementary Note 2

Nanofibrous mesh-integrated MEMS microphones

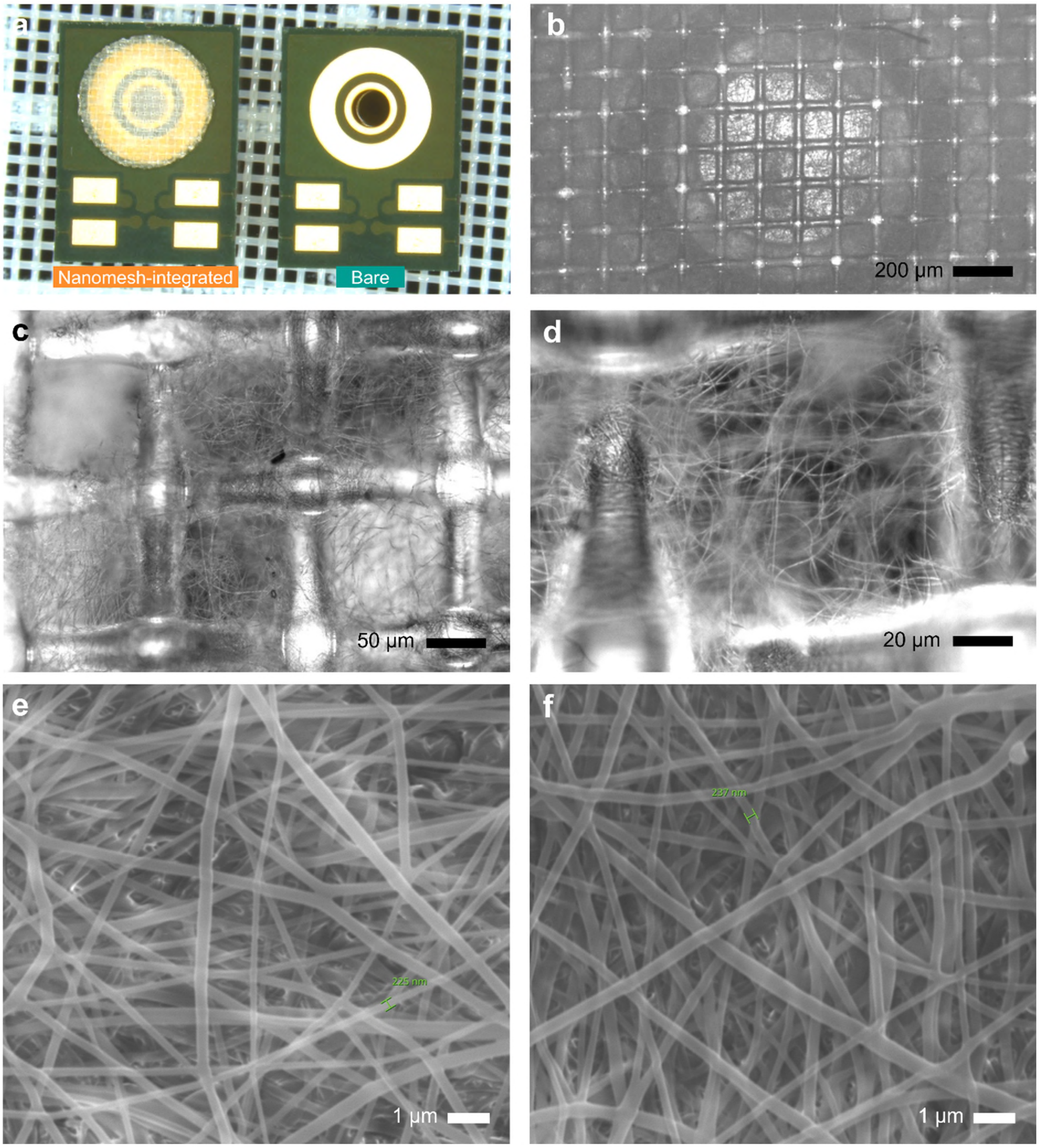

Supplementary Figure 2. Nanofibrous mesh-integrated MEMS microphone. a Photograph of a nanofibrous mesh-integrated microphone in comparison to its bare counterpart. **b–d** Optical microscopy images of PI nanofibrous mesh from SAATI SPA at different magnification views. **e, f** Scanning electron microscopy (SEM) images of PI nanofibrous mesh showing its morphology and size.

Supplementary Note 3

Lumped-element-based simulation of mesh-protected microphone

Supplementary Table 1. Parameters for lumped-element-based simulation of mesh-protected microphone. These parameters are used in the equivalent circuit model shown in **Figure 3c** in the main article**.**

Parameter	Value	Unit
R_{MEMS}	2.16×10^{7}	Pa s/m³
L_{mem}	1170.36	kg/m⁵
C_{mem}	6.55×10^{-15}	m³/Pa
R_{Vent}	1.36×10^{11}	Pa s/m³
$C_{0,MEMS}$	4	pF
η_{el}	18.63	m×V/Pa
R_{Port}	1.51×10^{6}	Pa s/m³
L_{Port}	1064.64	kg/m⁵
C_{Port}	4.23×10^{-15}	m³/Pa
C_{BV}	4.32×10^{-14}	m³/Pa

Supplementary Note 4

Electroacoustic measurement setup

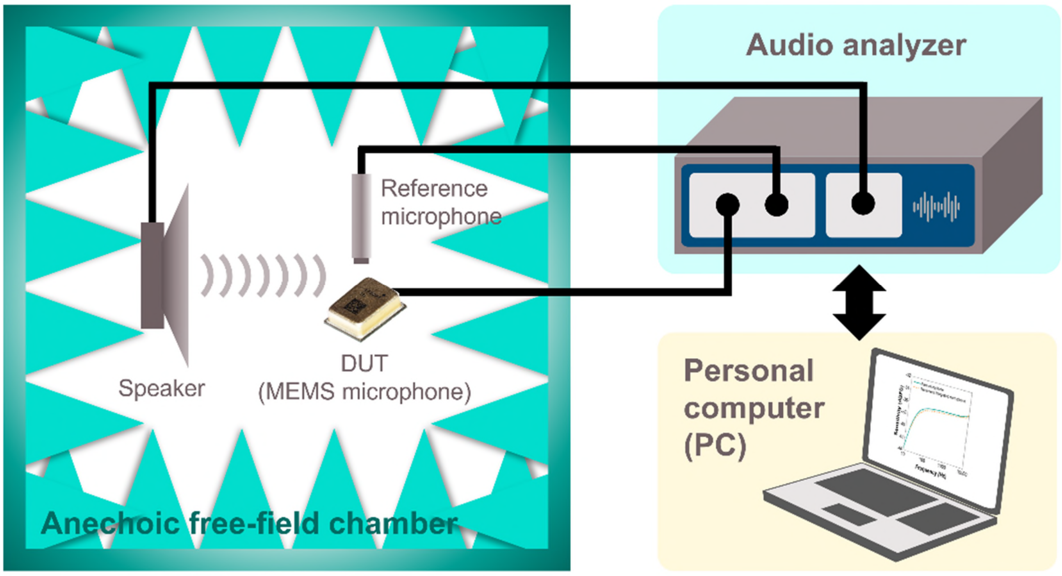

Supplementary Figure 3. Electroacoustic measurement setup in an anechoic free-field chamber (foam-filled cubic enclosure). The device-under-test (DUT), which is a MEMS microphone, is placed close to a reference microphone. The distance between speaker and both microphones is set to be ~50 cm. An audio analyzer is employed to monitor the output voltages from the DUT and the reference microphone as well as to actuate the loudspeaker. All measurements are controlled and stored using a built-in software installed in a personal computer (PC).

Supplementary Note 5

Pressure tests of nanofibrous meshes

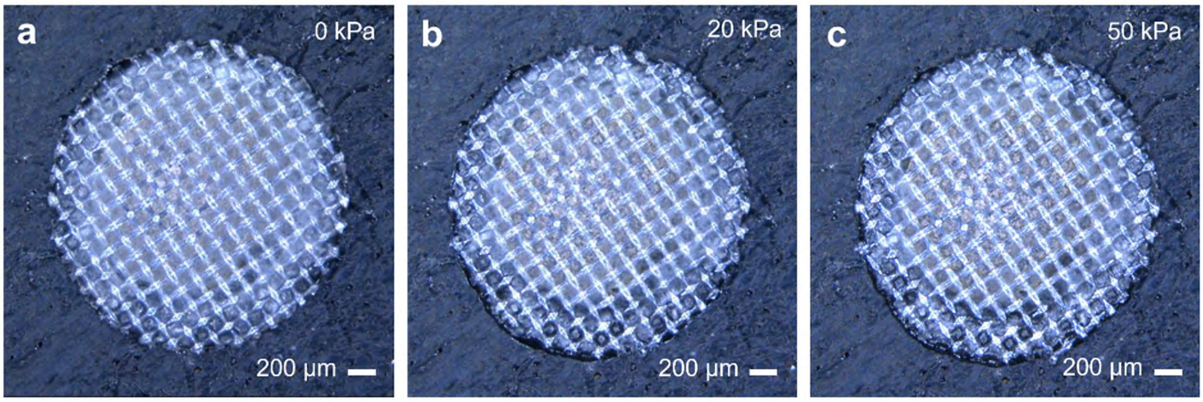

Supplementary Figure 4. Static pressure test results of nanofibrous meshes. Optical microscopy images of nanofibrous meshes **a** before and after static pressure tests at **b** 20 and **c** 50 kPa. In both cases, after pressure has been applied, the polyimide (PI) nanofibers appear to be unaltered.

Supplementary Note 6

<u>Hair drop tests of nanomesh-integrated MEMS microphones</u>

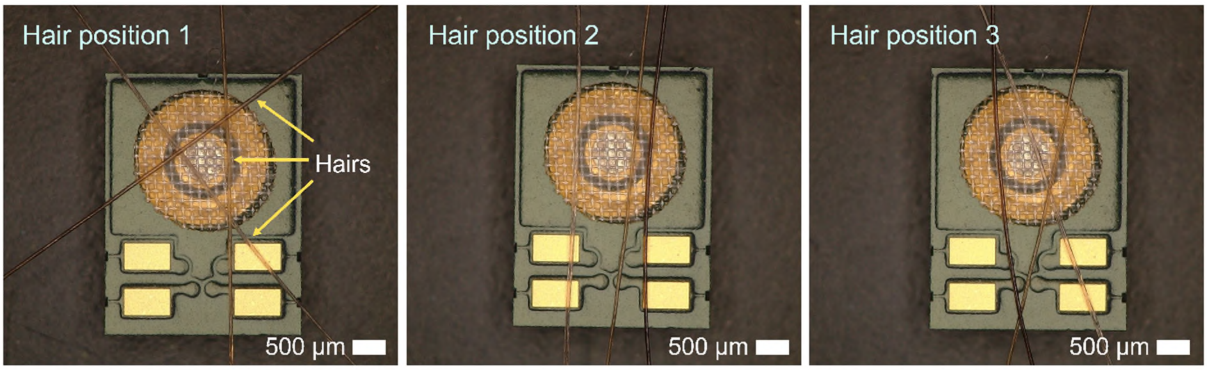

Supplementary Figure 5. Hair drop test results of nanomesh-integrated MEMS microphones. A few hairs were accurately dropped onto a nanofibrous mesh-integrated MEMS microphone. The dropping process was conducted with a height/gap of ~0.5 cm from the upper surface of the nanomesh. Different positions of hairs (i.e., hair positions 1 – 3) were attempted to prove the protection ability of mesh towards hairs from altered directions.

Supplementary Note 7

Wettability tests of nanomesh-integrated MEMS microphones

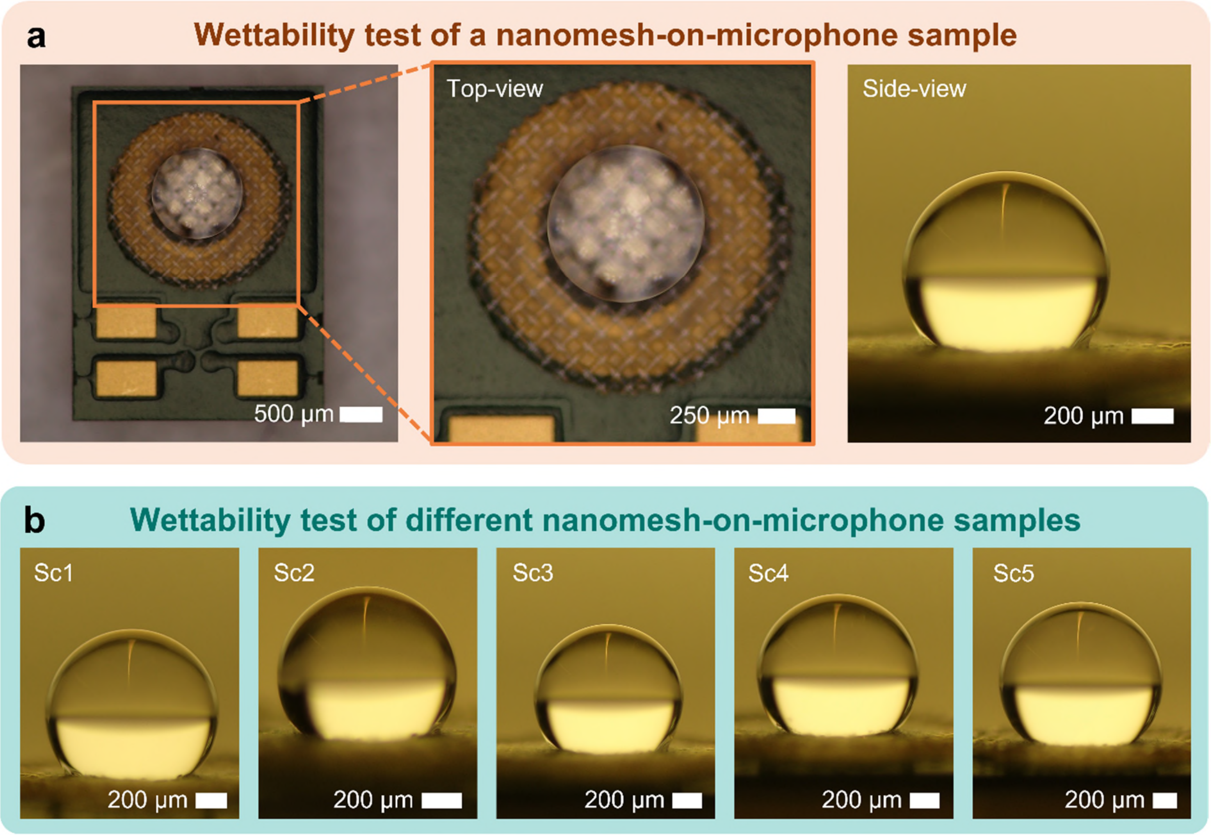

Supplementary Figure 6. Wettability test results of nanomesh-integrated MEMS microphones. a Water contact angle (WCA) measurements conducted to evaluate the wettability of a nanofibrous mesh attached on a microphone. **b** Five different PI-based nanomesh-on-microphone samples (Sc1 – Sc5) were measured resulting in WCA values of (132.8 ± 5.1)°. These results demonstrated the intrinsic surface hydrophobicity of the PI-based nanomesh.

Supplementary Note 8

Dust microparticle tests of nanomesh-integrated MEMS microphones

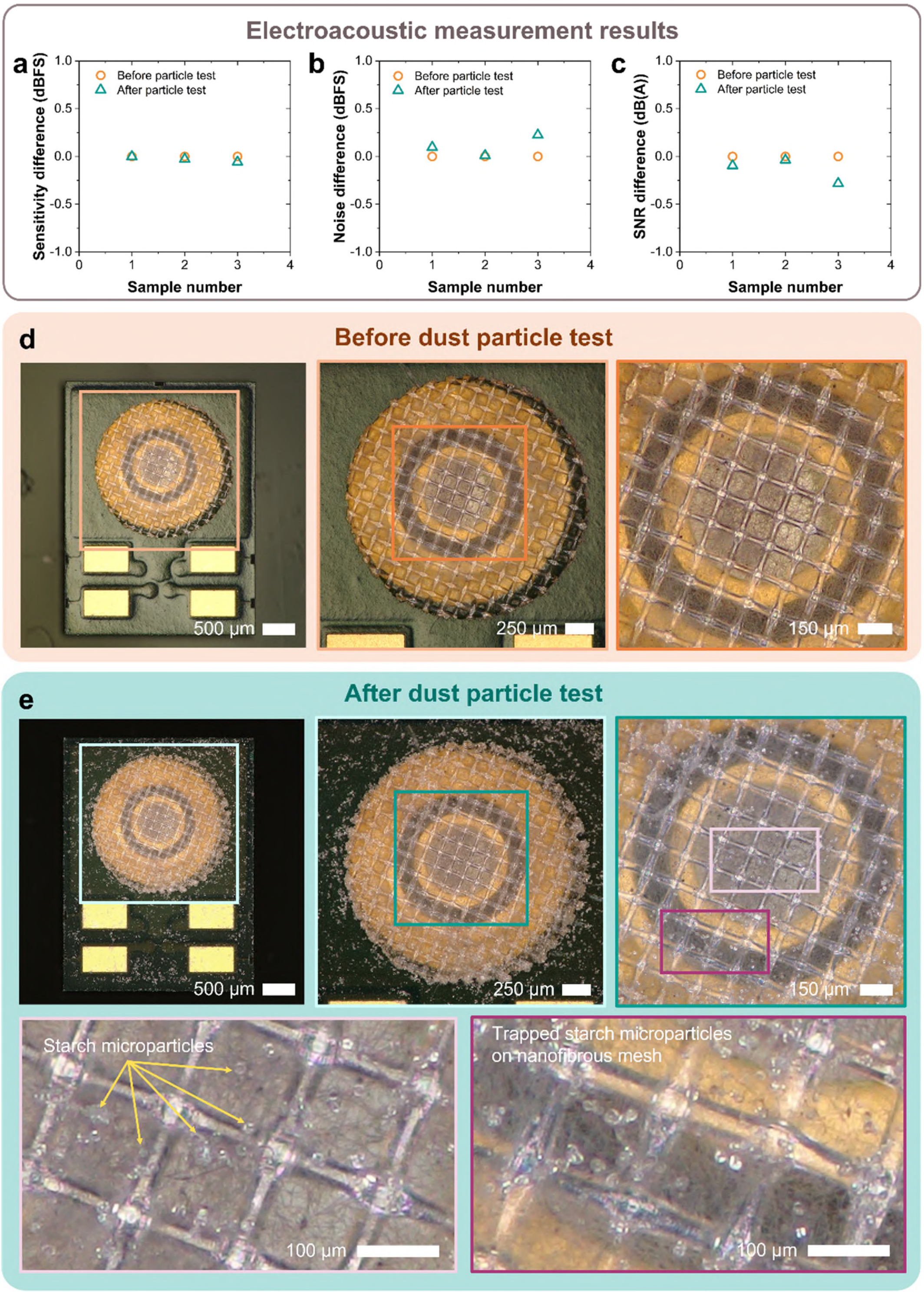

Supplementary Figure 7. Dust-microparticle test results of nanomesh-integrated MEMS microphones. Differences in **a** sensitivity, **b** noise, and **c** signal-to-noise ratio (SNR) values before and after dust-microparticle tests were used to evaluate the performances of nanomesh-integrated microphones. Typically obtained optical microscopy images of nanomesh-integrated microphone **d** before and **e** after dust-microparticle test. Polydisperse native starch particles with sizes of 0.9 – 61 μm were employed as test dusts.
